# Role of Synaptosomal-Associated Protein 25 Gene in Clinical Response to Methylphenidate Among Turkish Pediatric ADHD Cohort

**DOI:** 10.3390/children13101412

**Published:** 2026-10-15

**Authors:** Zeynep Gizem Todurga-Seven, Kubra Cigdem Pekkoc-Uyanik, Muhammet Fatih Akdemir, Melik Yigit Bayindir, Aybike Aydin Okuyan, Mahmut Cem Tarakcioglu, Sibel Ozyazgan, Hafize Uzun

**Affiliations:** 1 Department of Medical Pharmacology, Cerrahpasa Faculty of Medicine, İstanbul University-Cerrahpasa, İstanbul 34098, Türkiye; zeynep.seven@iuc.edu.tr (Z.G.T.-S.); muhammet.akdemir@iuc.edu.tr (M.F.A.); melik.bayindir@iuc.edu.tr (M.Y.B.); sibel.ozyazgan@iuc.edu.tr (S.O.); 2 Department of Medical Biology, Medical Faculty, Haliç University, İstanbul 34060, Türkiye; kubrapekkoc@halic.edu.tr; 3 Department of Child and Adolescent Psychiatry, Cerrahpasa Faculty of Medicine, İstanbul University-Cerrahpasa, İstanbul 34098, Türkiye; aybike.aydin@iuc.edu.tr (A.A.O.); mc.tarakcioglu@iuc.edu.tr (M.C.T.); 4 Department of Biochemistry, Faculty of Medicine, İstanbul Atlas University, İstanbul 34408, Türkiye

**Keywords:** ADHD, methylphenidate, *SNAP-25*, next-generation sequencing

## Abstract

**Highlights:**

**What are the main findings?**

- In children and adolescents with ADHD, the *SNAP-25* rs362998 T allele was associated with a positive response to methylphenidate (MPH), as assessed using the Conners’ Parent Rating Scale (CPRS).
- Targeted next-generation sequencing identified a previously unreported *SNAP-25* variant, c.*54T > C, in the 3′ untranslated region.

**What are the implications of the main findings?**

- Genetic variation in *SNAP-25* may contribute to individual differences in methylphenidate response, suggesting a potential role for synaptic mechanisms in ADHD treatment outcomes.
- These findings highlight the potential contribution of *SNAP-25* to interindividual variability in the response to MPH; however, its significance for personalized ADHD treatment requires further study in larger, independent cohorts.

**Abstract:**

**Introduction:** Methylphenidate (MPH) is a first-line treatment for attention-deficit/hyperactivity disorder (ADHD). MPH blocks dopamine and norepinephrine transporters, increasing synaptic catecholamine levels. Neurotransmitter release depends on presynaptic vesicle docking and fusion mediated by synaptosomal-associated protein 25 kDa (SNAP-25), a target-soluble NSF attachment protein receptor (SNARE) protein encoded by the *SNAP-25* gene. This study aimed to investigate whether genetic variations in the *SNAP-25* gene contribute to variability in clinical response to MPH in pediatric patients with ADHD. **Methods:** Forty-two children and adolescents aged 6–16 years who presented to the outpatient clinic and were diagnosed with ADHD according to DSM-5-TR criteria were prospectively treated with MPH for two months. Clinical response was assessed using the Conners’ Parent Rating Scale (CPRS) and the Turgay DSM-based Disruptive Behavior Disorders Rating Scale (T-DSM-IV-S). *SNAP-25* exons were sequenced using targeted next-generation sequencing (tNGS). **Results:** Three common variants in *SNAP-25* (rs363006, rs362585, and rs362998) were analyzed. Based on CPRS-defined outcomes, a significant association was observed between the rs362998:C > T polymorphism and response to MPH. In the genotypic model, the CT genotype was more frequent among responders (34.78%) than non-responders (5.26%) (*p* = 0.026, FDR-adjusted *p* = 0.049). In the dominant model, CT + TT genotypes were more frequent in responders (*p* = 0.026, FDR-adjusted *p* = 0.049). The allelic model showed a higher frequency of the T allele in responders (*p* = 0.036, FDR-adjusted *p* = 0.049). Haplotype analysis showed that rs362585, rs362998, and rs363006 formed a single haplotype block. The GC haplotype (rs362585–rs362998) was more frequent among non-responders (*p* = 0.016), while the GCG haplotype showed a similar trend among non-responders, although it did not reach statistical significance (*p* = 0.058). However, no haplotype association remained statistically significant after FDR correction. A novel 3′UTR variant (c.*54T > C; chr20:10306251T > C) was also identified. **Conclusions:** These exploratory findings suggest a potential association between *SNAP-25* rs362998:C > T and MPH response in pediatric ADHD; however, the small sample size and wide confidence intervals warrant cautious interpretation and validation in larger, independent cohorts.

## 1. Introduction

Attention-deficit/hyperactivity disorder (ADHD) is a prevalent neurodevelopmental condition characterized by inattention, hyperactivity, and impulsivity [1]. While global prevalence is estimated at approximately 5.9% [2], multicenter studies in Türkiye have reported rates as high as 12.4% among school-aged children [3]. Methylphenidate (MPH) remains the gold-standard, first-line pharmacotherapy for managing these symptoms [4]. However, approximately 20% to 30% of patients exhibit a suboptimal therapeutic response or intolerable side effects. This significant interindividual variability underscores the need to identify pharmacogenetic factors associated with MPH treatment response [2].

MPH exerts its therapeutic effects primarily by inhibiting presynaptic dopamine (DA) and norepinephrine (NE) transporters, thereby enhancing catecholaminergic neurotransmission [5]. These mechanisms and interindividual differences in treatment response may be further influenced by interactions between pharmacological effects and patient genotypes. Accordingly, pharmacogenomic research seeks to explain why certain medications are more effective or better tolerated in specific genetic backgrounds [6]. To date, most pharmacogenomic markers identified for psychiatric medications are related to drug metabolism rather than mechanisms of action [7]. Although the US Food and Drug Administration (FDA) has not yet included pharmacogenomic biomarkers in the MPH label, extensive research has examined the impact of genetic variation on its efficacy, implicating multiple pathways involving monoaminergic signaling [8,9,10]. However, the efficiency of monoaminergic signaling is not determined solely by transporter activity but also by presynaptic mechanisms governing synaptic vesicle docking, fusion, and neurotransmitter exocytosis [11].

The synaptosomal-associated protein 25 (SNAP-25) is a core component of the SNARE complex and is essential for calcium-dependent synaptic vesicle fusion and neurotransmitter release [12]. This function is mediated through coiled-coil interactions with syntaxin-1 and synaptobrevin, enabling efficient presynaptic membrane fusion in response to Ca^2+^ signals. Complete absence of SNAP-25 results in a profound failure of synaptic communication, whereas reduced *SNAP-25* expression impairs SNARE complex assembly and vesicle fusion, ultimately leading to synaptic dysfunction [13]. Alterations in *SNAP-25* expression or function can disrupt synaptic efficiency and neuronal communication, processes that are highly relevant to the pathophysiology of ADHD [14].

Therefore, genetic variation in the *SNAP-25* gene may influence the downstream effects of MPH by regulating presynaptic neurotransmitter release, thereby affecting the magnitude of dopaminergic signal amplification achieved with stimulant therapy. Previous pharmacogenetic studies have suggested a potential association between genetic variation in the *SNAP-25* gene and MPH treatment response in children with ADHD. In particular, the rs3746544:T > G polymorphism has been investigated in relation to clinical response to MPH in pediatric ADHD populations, with evidence indicating that *SNAP-25* variants may contribute to interindividual differences in treatment responsiveness [15,16]. A meta-analysis evaluating multiple *SNAP-25* polymorphisms, including rs362998, also reported an association between rs3746544 and ADHD susceptibility, whereas no significant association was identified for rs362998 [17]. These findings concern disorder susceptibility rather than pharmacological response, and variants associated with ADHD risk may not necessarily predict MPH treatment response. Therefore, the potential role of *SNAP-25* variants in treatment response warrants further investigation.

Due to the limited research on *SNAP-25* variation and MPH treatment response, it remains unclear whether variants identified in Turkish children and adolescents are associated with differences in clinical improvement. Evidence concerning ADHD susceptibility does not resolve this treatment-response question, and the contribution of combinations of *SNAP-25* variants also requires investigation.

This exploratory study aimed to investigate associations between *SNAP-25* genetic variation and response to 2 months of MPH treatment in a Turkish pediatric cohort diagnosed with ADHD according to Diagnostic and Statistical Manual of Mental Disorders, Fifth Edition, Text Revision (DSM-5-TR) criteria. Exonic regions and exon–intron boundaries were examined using targeted next-generation sequencing (tNGS), and common variants were evaluated individually and in exploratory haplotype analyses. The primary outcome measures were responder status at 2 months, assessed separately using the Conners’ Parent Rating Scale (CPRS) and the Turgay DSM-based Disruptive Behavior Disorders Rating Scale (T-DSM-IV-S). Response was defined as a reduction from baseline in the summed CPRS ADHD-related item score (items 4, 5, 11, and 13) and, separately, a ≥20% reduction in the summed score of the first 18 T-DSM-IV-S items.

## 2. Methods

### 2.1. Ethics Statement

The project was accepted by the İstanbul University-Cerrahpaşa Clinical Research Ethics Committee (Reference number 2025/429, 7 July 2025) and complied with the Declaration of Helsinki. Written informed consent was obtained from the parents of all participating children. Peripheral whole-blood samples from all participants were obtained in the Departments of Medical Pharmacology and Child and Adolescent Psychiatry at İstanbul University-Cerrahpaşa, Cerrahpaşa Faculty of Medicine (İstanbul, Türkiye).

### 2.2. Study Design

This pilot study was conducted as a single-center, prospective, observational pharmacogenetic investigation in the Departments of Medical Pharmacology and Child and Adolescent Psychiatry at İstanbul University-Cerrahpaşa, Cerrahpaşa Faculty of Medicine (İstanbul, Türkiye). Clinical evaluation and methylphenidate treatment were carried out in the Department of Child and Adolescent Psychiatry, whereas experimental procedures and molecular analyses were performed in the Department of Medical Pharmacology. The study sample consisted of 42 children and adolescents aged 6–16 years who presented to the outpatient clinic and were diagnosed with ADHD according to DSM-5-TR criteria [1]. The study, including participant recruitment and data collection, was conducted between October and November 2025.

Inclusion criteria were defined as being between 6 and 16 years of age, having a primary diagnosis of ADHD according to DSM-5-TR criteria, being treatment-naive for MPH, and having at least one literate parent with adequate Turkish comprehension. Baseline exclusion criteria consisted of major neurodevelopmental or psychiatric disorders, epilepsy, fragile X syndrome, serious chronic medical illness, prenatal substance exposure, and fetal alcohol syndrome. A total of 66 eligible pediatric patients diagnosed with ADHD and their parents were approached for study participation. Of these, 10 declined participation, primarily because of reluctance to undergo blood sampling for genetic analysis or scheduling constraints. Consent was obtained for the remaining 56 patients. Before treatment initiation, two patients were excluded because they were taking concomitant medications with clinically significant interactions with MPH. Following these exclusions, 54 patients initiated MPH treatment. During the 2-month follow-up period, 12 patients were excluded from the final analysis: three voluntarily discontinued medication, seven were lost to follow-up or declined to complete the second-month rating scales, and two discontinued treatment because of intolerable adverse effects. Ultimately, 42 patients completed the full 2-month evaluation and were included in the final pharmacogenetic and clinical response analyses.

Following diagnosis, psychoeducation about ADHD and MPH treatment was provided to the child and family, and those who agreed to participate completed a structured sociodemographic data form. Age, sex, height, weight, school performance, and core clinical characteristics were recorded systematically.

Symptom severity was additionally rated with the Turkish version of the T-DSM-IV-S, an instrument originally developed and published in English [18] and validated for use in Turkish children and adolescents [19]. Its items correspond directly to the DSM symptom criteria (inattention, 9 items; hyperactivity–impulsivity, 9 items; oppositional defiant disorder, 8 items; conduct disorder, 15 items), and the first 18 items were used to derive ADHD symptom severity. Also, the CPRS (Conduct Problems, Impulsivity–Hyperactivity, Learning Problems, Anxiety and Psychosomatic Complaints) was administered following MPH treatment to assess changes in symptom severity [20]. Parent-completed scales were selected to facilitate consistent assessment at baseline and follow-up in this outpatient study. The assessment focused on treatment-related changes in core ADHD symptoms. Accordingly, the first 18 items of the T-DSM-IV-S, covering 9 inattention and 9 hyperactivity/impulsivity symptoms, were used to assess treatment response; items assessing oppositional defiant and conduct disorder symptoms were not included in the response score.

Treatment response was evaluated separately for each scale in the 42 patients included in the final analysis. For the T-DSM-IV-S, response was defined as a ≥20% reduction from baseline in the summed score of the first 18 items, yielding 20 responders and 22 non-responders. For the CPRS, response was defined as a reduction from baseline in the summed score of the ADHD-related items (items 4, 5, 11, and 13), yielding 23 responders and 19 non-responders.

MPH treatment was initiated in accordance with national and international guideline recommendations and clinical needs, using short- or long-acting oral formulations [21]. Dose titration was performed by the treating clinician based on body weight, symptom control, and tolerability [22]. Medication selection and dose adjustment were not altered for research purposes; treatment was delivered within the framework of routine clinical practice.

### 2.3. Genomic DNA Isolation and NGS Library Preparation

Genomic DNA was extracted from peripheral blood samples collected in EDTA-containing tubes using the Genomic DNA Column Kit (Jena Bioscience, Jena, Germany) in accordance with the manufacturer’s instructions. DNA quantity and quality were assessed fluorometrically using the Qubit 3.0 Fluorometer (Invitrogen, Carlsbad, CA, USA). All exons of the *SNAP-25* gene, together with exon–intron junction regions, were amplified by polymerase chain reaction (PCR) using a MiniAmp Thermal Cycler (Thermo Fisher Scientific, Waltham, MA, USA). For targeted sequencing, an amplicon-based library preparation strategy was implemented. Gene-specific primers were manually designed for each exon and boundary region, and PCR products obtained from individual samples were subsequently subjected to indexing. The resulting PCR amplicons were indexed, pooled, and sequenced using the MiSeq system (Illumina, Inc., San Diego, CA, USA). Sequencing was performed on the MiSeq platform (Illumina, San Diego, CA, USA). This tNGS approach provided comprehensive coverage of the selected regions, achieving a minimum read depth of 100× across all targeted bases.

### 2.4. Analyzing Raw Data

Raw reads were assessed with FastQC, trimmed using Trimmomatic, and aligned to the Genome Reference Consortium Human Build 38 (GRCh38) genome with BWA-MEM via the DRAGEN platform (version 4.3.6.). The generated CRAM file stored a Phred-scale quality (Q) score for each nucleotide mapped to the reference, in accordance with the process described in previous studies [23,24]. Variants were called using GATK HaplotypeCaller and filtered by standard criteria, retaining only those with ≥30× depth and a “PASS” status. Annotations were carried out with VEP (version 115.2), and results were reviewed in Integrative Genomics Viewer (IGV, version 3.8.3). Rare and potentially functional variants were prioritized and interpreted in clinical data.

### 2.5. Statistics

Statistical analyses were performed using the GraphPad Prism version 10.6.1 software program. The normality of age was assessed using the Shapiro–Wilk test. Since age was not normally distributed, comparisons between responsive and non-responsive and analyses of the association between age and genotype were performed using the Mann–Whitney U test. Differences in sex and body mass index (BMI) categories between groups, as well as their associations with genotype, were assessed using Fisher’s exact test. Statistical analyses between the study groups and genotypes were performed using Fisher’s exact test. Data are expressed as mean ± standard deviation (SD) or as number and percentage. *p* < 0.05 values were considered statistically significant. Allele and genotype distributions were calculated, including Hardy–Weinberg equilibrium (HWE) estimates. Deviations from HWE were examined using an online tool developed by Michael H. Court (2005–2008). Haplotype structure and linkage disequilibrium (LD) were analyzed using the Haploview program, evaluating both individual and combined marker relationships. The Benjamini–Hochberg procedure was used to control the false discovery rate (FDR) separately for each SNP across the genotypic, dominant, recessive, and allelic tests. FDR-adjusted *p* < 0.05 was considered statistically significant.

## 3. Results

The final analysis included 42 children and adolescents who completed the two-month follow-up. Baseline demographic and BMI characteristics, separately stratified by CPRS- and T-DSM-IV-S-defined treatment response, and their associations with *SNAP-25* genotypes are presented in Table 1.

Analysis using tNGS of the SNAP-25 gene in children diagnosed with ADHD identified a total of five variants, including both common and rare polymorphisms (Table 2).

**Figure 1:**
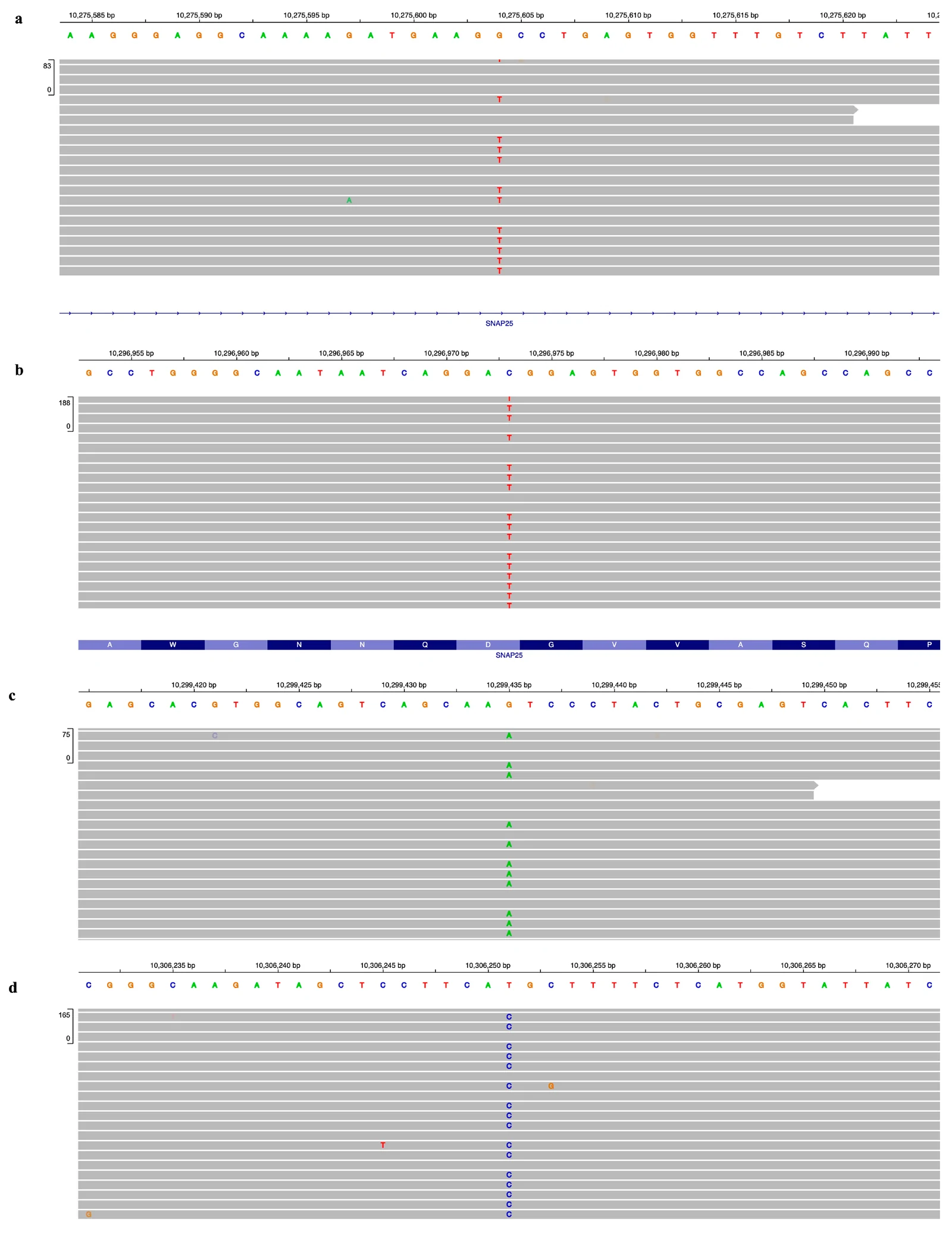
Integrative Genomics Viewer (IGV) read alignments for *SNAP-25* gene common variants: (**a**) rs362585:G > T (c.72 + 41G > T), (**b**) rs362998:C > T (c.330C > T; p.Asp110=), (**c**) rs363006:G > A (c.552 + 23G > A), and (**d**) the novel 3′UTR variant c.*54T > C (chr20:10306251T > C; GRCh38). Gray bars represent aligned sequencing reads, and colored letters indicate bases differing from the reference sequence.

Four of the detected variants were previously reported in the single-nucleotide polymorphism database (dbSNP) and classified as benign according to American College of Medical Genetics and Genomics (ACMG) criteria. These included a 5′ untranslated region (UTR) variant (c.-34C > A, rs11547872), two intronic variants (c.72 + 41G > T, rs362585; c.552 + 23G > A, rs363006), and one synonymous exonic variant (c.330C > T, rs362998; p.Asp110=). The minor allele frequencies (MAFs) of these variants ranged from 0.03 to 0.34 in the highest reported population datasets, indicating that they represent common polymorphisms. Genotype distributions of all analyzed variants were consistent with HWE, and no statistically significant deviations were observed (*p* > 0.05). In addition, a novel 3′UTR variant (c.*54T > C; chr20:10306251 T > C) was identified. This variant was absent from dbSNP at the time of analysis and has not been reported in population databases. Its location within the 3′UTR suggests a possible regulatory role; however, functional studies have not yet been conducted to clarify its biological relevance. Pathogenic variants were not identified in the coding regions of *SNAP-25* in this cohort. Figure 1 presents Integrative Genomics Viewer (IGV) read alignments for three previously reported *SNAP-25* variants (panels a–c) and the novel 3′UTR variant (panel d). Gray bars represent aligned sequencing reads, and colored bases indicate differences from the reference sequence. These views provide sequencing support for the identification of these variants in the study cohort.

**Figure 2:**
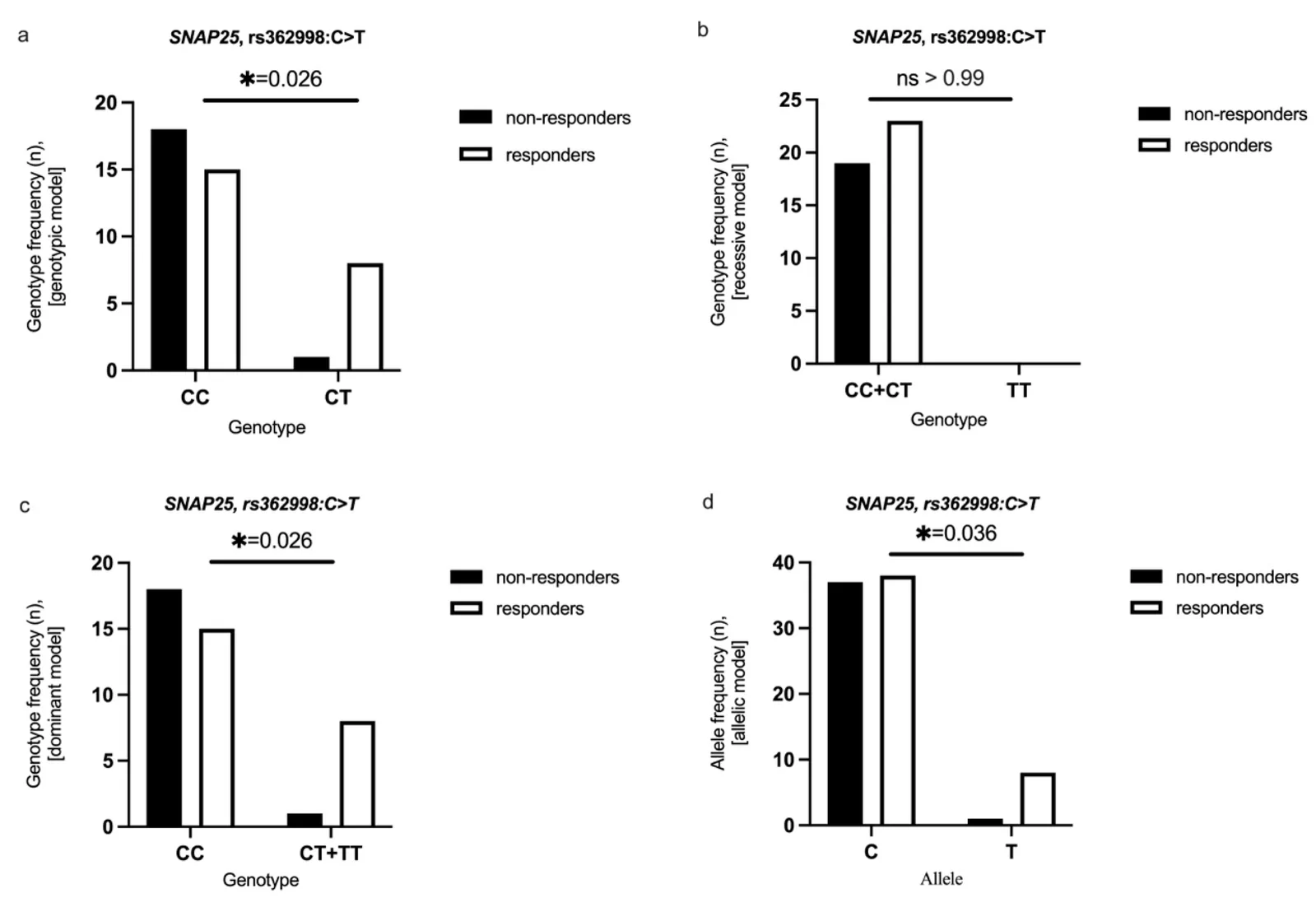
Association between the *SNAP-25* rs362998:C > T polymorphism and methylphenidate (MPH) treatment response in ADHD patients assessed by the CPRS: (**a**) A significant association was observed under the genotypic model (*p* = 0.026; OR = 9.60, 95% CI: 1.39–111.5, FDR-adjusted *p* = 0.049). (**b**) No significant association was detected under the recessive model (non-sginificant (ns), *p* > 0.99). (**c**) A significant association was identified under the dominant model (*p* = 0.026; OR = 9.60, 95% CI: 1.39–111.5, FDR-adjusted *p* = 0.049). (**d**) The allelic model demonstrated a significantly higher frequency of the T allele in the responder group (*p* = 0.036; OR = 7.79, 95% CI: 1.05–88.5, FDR-adjusted *p* = 0.049). Fisher’s exact test was used for all comparisons. An asterisk (*) indicates statistical significance (*p* < 0.05); ns, not significant.

According to the T-DSM-IV-S scale, no statistically significant association between *SNAP-25* variants and treatment response was observed. In contrast, according to the CPRS, statistically significant associations were detected for rs362998:C > T, whereas rs362585:G > T did not reach statistical significance. Genotype and allele distributions were analyzed under genotypic, dominant, recessive, and allelic models using Fisher’s exact test. In the analysis of the *SNAP-25* gene rs362998:C > T polymorphism, a statistically significant association was observed between genotype distribution and treatment response under the genotypic model (*p* = 0.026, OR = 9.6, 95% CI: 1.388–111.5, FDR-adjusted *p* = 0.049). The CT genotype was more frequent among responders (34.78%) compared to non-responders (5.26%). Carriers of the CT genotype showed increased odds of treatment response compared with CC homozygotes (Figure 2a). Under the dominant model (CT + TT vs. CC), the association remained statistically significant (*p* = 0.026, OR = 9.6, 95% CI: 1.388–111.5, FDR-adjusted *p* = 0.049), indicating that carriers of the T allele had increased odds of treatment response compared with CC homozygotes (Figure 2c). In the allelic model, the T allele frequency was significantly higher in responders (17.39%) than in non-responders (2.63%) (*p* = 0.036, OR = 7.789, 95% CI: 1.047–88.51, FDR-adjusted *p* = 0.049), indicating increased odds of treatment response (Figure 2d). In contrast, no statistically significant association was detected under the recessive model (TT vs. CC + CT) (*p* > 0.05) (Figure 2b).

**Figure 3:**
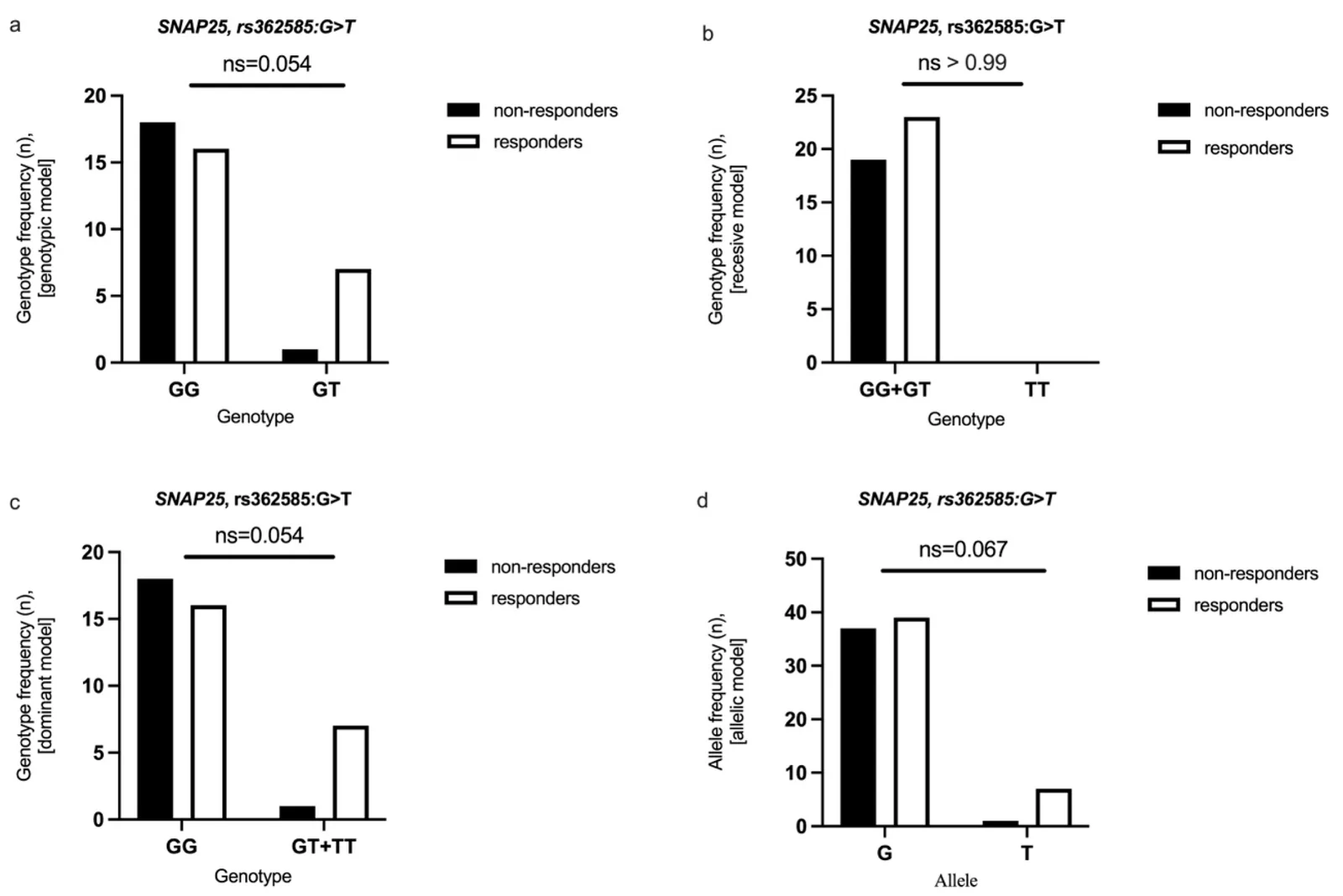
Association between *SNAP-25* rs362585:G > T polymorphism and methylphenidate (MPH) treatment response in ADHD patients assessed by the CPRS: (**a**) A borderline association was observed under the genotypic model (*p* = 0.054; OR = 7.88, 95% CI: 1.08–92.85). (**b**) No significant association was detected under the recessive model (*p* > 0.99). (**c**) A borderline association was identified under the dominant model (*p* = 0.054; OR = 7.88, 95% CI: 1.08–92.85). (**d**) The allelic model demonstrated a borderline enrichment of the T allele in the responder group (*p* = 0.067; OR = 6.64, 95% CI: 1.07–76.56). Fisher’s exact test was used for all comparisons. ns, not significant.

In the analysis of the rs362585:G > T variant, no statistically significant association was observed under the genotypic model, although the result approached significance (*p* = 0.054, OR = 7.875, 95% CI: 1.081–92.85). The GT genotype was more frequent among responders than non-responders (30.43% vs. 5.26%), suggesting a potential association with increased treatment response among GT carriers (Figure 3a). Consistently, under the dominant model (GT + TT vs. GG), T allele carriers were more common in the responsive group, demonstrating a similar borderline association (*p* = 0.054, OR = 7.875, 95% CI: 1.081–92.85) (Figure 3c). In the recessive model (TT vs. GG + GT), no significant association was detected (*p* > 0.05), in line with the absence of the TT genotype in the cohort (Figure 3b). Similarly, allelic analysis showed a higher frequency of the T allele in responders than in non-responders (15.22% vs. 2.63%), although this difference did not reach statistical significance (*p* = 0.067, OR = 6.641, 95% CI: 1.070–76.56) (Figure 3d). The rs362585:G > T variant did not reach statistical significance; however, the observed genotype distribution patterns and elevated odds ratios (ORs) suggest a potential association between the T allele and improved treatment response. These findings warrant cautious interpretation considering the limited sample size and the wide confidence intervals.

### Haplotype Analysis of SNAP-25 Gene Variants and MPH Response

**Figure 4:**
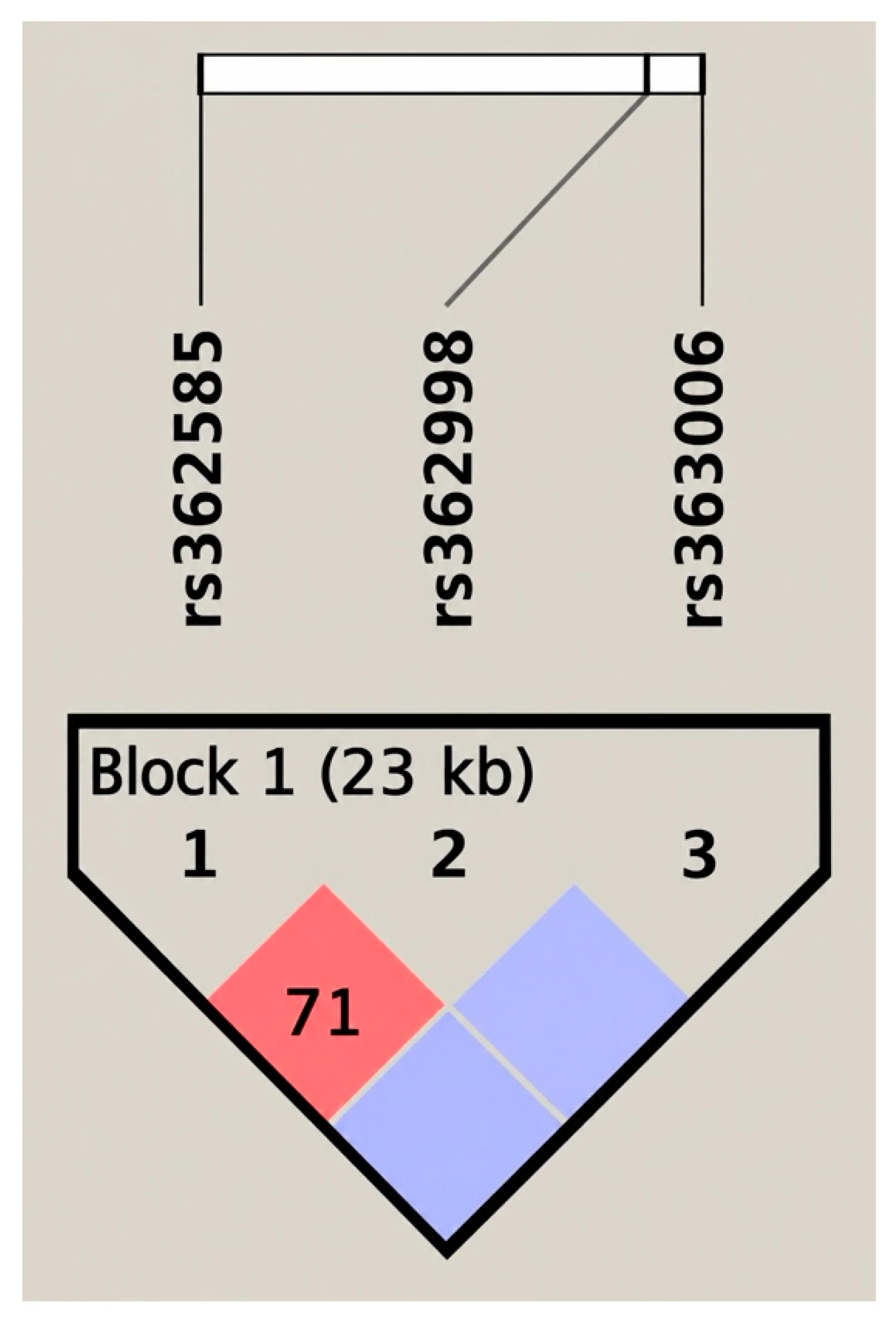
*SNAP-25* gene variations’ LD scheme. Pink/red cells indicate D′ < 1 and LOD ≥ 2; blue cells indicate D′ = 1 and LOD < 2. LD, linkage disequilibrium; LOD, logarithm of odds.

Haplotype analysis was performed for three polymorphisms in the *SNAP-25* gene: rs362585:G > T, rs362998:C > T, and rs363006:G > A (Table 3). LD analysis demonstrated that these variants were located within a single haplotype block spanning approximately 23 kb (Figure 4), with moderate LD observed particularly between rs362585 and rs362998. Haplotype distributions were compared between responder and non-responder groups, classified according to CPRS in 42 children diagnosed with ADHD.

In single-marker analysis, the G allele of rs362585 (M1) and the C allele of rs362998 (M2) showed borderline associations with treatment response (χ^2^ = 3.825, *p* = 0.050 for both). No significant association was observed for rs363006 (M3) (*p* = 0.959). In two-marker haplotype analysis, the GC haplotype (M1 + M2) was significantly more frequent in the non-responder group compared to the responder group (0.973 vs. 0.803), demonstrating a statistically significant association with reduced MPH response (χ^2^ = 5.714, *p* = 0.016). Other M1 + M2 haplotypes (TT, TC, and GT) did not reach statistical significance. For the M1 + M3 and M2 + M3 combinations, the TG haplotype showed a borderline association with treatment response (χ^2^ = 3.825, *p* = 0.050), being more frequent in the responder group (0.152) than in the non-responder group (0.026). Other haplotypes within these combinations were not statistically significant. The three-marker (M1 + M2 + M3) GCG haplotype further supports the possibility of cumulative genetic effects within this locus (χ^2^ = 3.577, *p* = 0.058), being more common in the non-responder group (0.868) compared to the responder group (0.694). However, this did not reach statistical significance. The remaining three-marker haplotypes (GCA, TTG, TCG, and GTG) were not significantly associated with MPH response (Table 4). However, none of the haplotype associations remained statistically significant after FDR correction.

The haplotype analysis indicates that specific combinations of rs362585 and rs362998 alleles, particularly the GC haplotype, may be associated with reduced response to MPH treatment in children with ADHD.

## 4. Discussion

ADHD is a neurodevelopmental condition for which MPH remains the prescribed first-line pharmacological treatment [25]. Despite its well-established efficacy, a substantial proportion of patients exhibit variable therapeutic response and tolerability, highlighting the need to better understand the biological factors underlying this heterogeneity. Genetic determinants, particularly those involved in synaptic transmission and dopaminergic regulation, have been proposed as potential contributors to differential stimulant response. The present study evaluated the association between common *SNAP-25* gene variants and MPH treatment outcomes, with treatment response defined as improvement in CPRS and T-DSM-IV-S scores. In our study, approximately 50% of patients were classified as non-responders. The higher proportion of non-responsiveness compared with previous reports may reflect differences in response assessment criteria, the relatively short treatment period, and sampling variability associated with the modest sample size.

The core SNARE complex in neurotransmission consists of SNAP-25, Syntaxin-1A, and vesicle-associated membrane protein (VAMP) (1 or 2). SNAP-25 forms a heterodimer with the presynaptic membrane protein Syntaxin-1A, which then binds to VAMP to assemble the SNARE complex, bringing the vesicle and plasma membranes together. Calcium influx activates synaptotagmin (Syt I or II), which interacts with the SNARE complex to trigger membrane fusion and neurotransmitter release [26]. Due to its central role in neurotransmitter release, genes encoding components of the SNARE complex and their regulatory proteins have been extensively investigated in psychiatric disorders. Variants within these genes have been proposed to influence synaptic efficiency, neuronal excitability, and network connectivity, thereby contributing to the pathophysiology of neurodevelopmental and mood disorders. In particular, *SNAP-25* has attracted considerable attention as a candidate gene, given its involvement in dopaminergic and glutamatergic signaling pathways that are frequently implicated in conditions such as ADHD, schizophrenia, and bipolar disorder [27]. In conclusion, several recent studies have shown a close association between *SNAP-25* gene polymorphisms and ADHD [28,29,30].

Previous studies have shown associations between common *SNAP-25* variants such as rs3746544 and rs1051312 and ADHD symptom severity [28,31,32]. However, these variants were not commonly observed in our population. In this research, three common *SNAP-25* variants (rs363006, rs362585, and rs362998) were evaluated in relation to MPH treatment response. Among the *SNAP-25* polymorphisms investigated, the rs362998:C > T variant was associated with improved MPH treatment response in the CPRS-defined analysis. Specifically, carriers of the T allele were more frequently represented in the responder group. However, the wide confidence interval indicates substantial uncertainty in the estimated effect size, limiting conclusions about the magnitude and clinical relevance of this association. Previous genetic studies have reported associations between several *SNAP-25* variants and ADHD susceptibility, including rs363006 and rs362998. Over-transmission of the C allele of rs362998 in ADHD families has been reported [33], whereas a significant association between rs363006 and ADHD diagnosis was described in an independent cohort [34]. Interestingly, within the current study, a statistically significant association between these *SNAP-25* variants and treatment response was not observed when evaluated according to the T-DSM-IV-S scores. The discrepancy between CPRS- and T-DSM-IV-S-based findings may be explained by several factors. First, treatment response according to the CPRS was determined using only four ADHD-related items, whereas the T-DSM-IV-S response classification was based on a broader set of ADHD symptom items. Therefore, the two scales may not capture treatment-related changes in the same manner. Second, although both instruments assess ADHD symptoms, they differ in their structure and primary focus, potentially resulting in different sensitivities to clinical improvement. Third, the responder and non-responder groups were not identical across the two outcome measures, indicating that some patients were classified differently depending on the assessment instrument used. Finally, the relatively small sample size may have further limited the ability to detect consistent pharmacogenetic effects across different outcome measures. Therefore, these findings should be interpreted cautiously until replicated in larger independent cohorts.

In the present study, haplotype analysis was performed on three *SNAP-25* polymorphisms (rs362585, rs362998, and rs363006). Among the evaluated combinations, the GC haplotype formed by rs362585 and rs362998 was significantly more frequent in the non-responder group, suggesting an association with reduced MPH response. The GC haplotype showed a nominal association with reduced MPH treatment response; however, this association did not remain statistically significant after FDR correction and should be interpreted as an exploratory finding. The observed pattern for the three-marker GCG haplotype further supports the possibility of cumulative or interactive genetic effects within this locus.

To our knowledge, there are no previously published studies that have specifically conducted haplotype analysis on the same combination of *SNAP-25* polymorphisms investigated in the present study (rs362585, rs362998, and rs363006). Hawi et al. performed haplotype analyses including rs6108461, rs362990, and rs362998 and demonstrated that the ADHD-associated haplotype was linked to reduced *SNAP-25* expression in post-mortem inferior frontal gyrus tissue [35]. Meta-analyses of *SNAP-25* polymorphisms support a role for some variants (e.g., rs3746544) in ADHD risk, but they have not reported haplotype effects for rs362585 and rs363006 [17]. The present study offers novel haplotypic evidence regarding the contribution of *SNAP-25* to interindividual variability in MPH treatment response among children with ADHD.

### 4.1. Strengths and Limitations

The prospective design, inclusion of patients who were treatment-naive for MPH, and targeted sequencing of *SNAP-25* exons and exon–intron boundaries represent key strengths of this study. The assessment of treatment response using two rating scales and the analysis of both individual variants and haplotypes provided complementary perspectives on the associations investigated.

The findings of this preliminary exploratory study should be interpreted in the context of several limitations. Primarily, the modest sample size may have restricted the precision of the effect estimates, reduced the statistical power required to detect genetic associations with smaller effect sizes, and increased the risk of both type I and type II errors. In addition, findings from small cohorts may be more susceptible to sampling variability, warranting cautious interpretation of the observed effect sizes. Furthermore, the single-center design and specific demographic characteristics of our Turkish pediatric cohort may limit the generalizability of the outcomes to other ethnic populations. Finally, the evaluation of clinical treatment response relied on parent-completed scales, specifically the CPRS and T-DSM-IV-S, without complementary teacher reports, age-appropriate self-reports, standardized clinician-rated outcome measures, or objective measures. A broader assessment of functioning was also not performed. These limitations restrict the evaluation of treatment-related changes across settings and beyond core ADHD symptoms. Therefore, the present findings should be considered preliminary and hypothesis-generating and need to be validated in larger and adequately powered multicenter cohorts to demonstrate their robustness and broader applicability.

### 4.2. Future Research

Future studies should seek to replicate the associations between *SNAP-25* variants and MPH response in larger, adequately powered, independent multicenter cohorts with diverse ancestry backgrounds and longer follow-up. Where feasible, plasma MPH concentrations could be measured at standardized post-dose time points. These measurements could support pharmacokinetic–pharmacodynamic analyses to assess whether associations between *SNAP-25* variants and treatment response persist after accounting for interindividual differences in drug exposure. Parent ratings should be complemented by teacher reports, clinician-rated measures, and assessments of functioning. Functional studies of the identified *SNAP-25* variants, including the novel 3′UTR variant, are also needed to clarify their biological effects and potential relevance to MPH response. Before clinical use, studies should determine whether adding *SNAP-25* genetic information to routine clinical assessment improves the prediction of MPH treatment response.

## 5. Conclusions

Due to significant variability in individual responses to MPH among patients with ADHD, identifying the genetic factors that contribute to differences in treatment outcomes remains a relevant clinical aim. In this exploratory study, we evaluated the potential role of common *SNAP-25* variants in modulating MPH treatment outcomes. Among the polymorphisms examined, the rs362998:C > T variant showed a potential association with therapeutic response, with the T allele being more prevalent in the responding group; however, the wide confidence interval warrants cautious interpretation, and further studies in larger independent cohorts are needed to clarify its clinical relevance. *SNAP-25* is a key mediator of synaptic vesicle docking and neurotransmitter release in dopaminergic and glutamatergic circuits. Genetic variation at this locus may modulate synaptic transmission dynamics, thereby influencing neuronal sensitivity to external stimuli. The findings should be interpreted cautiously due to the preliminary design of the study and limited sample size, but they support the hypothesis that variation in genes involved in synaptic mechanisms may contribute to pharmacological response variability in ADHD. Future studies should include larger, ethnically diverse cohorts and functional studies to elucidate the biological mechanisms underlying these relationships and to determine whether *SNAP-25* variants can serve as clinically informative biomarkers for individualized stimulant therapy strategies.

## Figures and Tables

**Table 1:** Baseline demographic and BMI characteristics of the 42 patients included in the final analysis, according to treatment response assessed separately using the CPRS and T-DSM-IV-S, and their associations with *SNAP-25* genotypes.

		T-DSM-IV-S			CPRS			rs362998:C > T			rs362585:G > T			rs363006:G > A		
Characteristics	Total (*n* = 42)	Responder (*n* = 20)	Non-Responder (*n* = 22)	*p* Value	Responder (*n* = 23)	Non-Responder (*n* = 19)	*p* Value	CC (*n* = 34)	CT (*n* = 8)	*p* Value	GG (*n* = 34)	GT (*n* = 8)	*p* Value	GG (*n* = 33)	GA (*n* = 9)	*p* Value
Gender, *n* (%)				0.499			0.323			0.195			0.668			1
Female	12	7 (35)	5 (22.7)		5 (21.7)	7 (36.8)		8 (23.5)	4 (50.0)		9 (26.5)	3 (37.5)		10 (30.3)	2 (22.2)	
Male	30	13 (65)	17 (77.3)		18 (78.3)	12 (63.2)		26 (76.5)	4 (50.0)		25 (73.5)	5 (62.5)		23 (69.7)	7 (77.8)	
Mean age ± SD (years)	8.29 ± 1.84	8.05 ± 1.85	8.50 ± 1.85	0.418	8.35 ± 2.01	8.21 ± 1.65	0.918	8.21 ± 1.82	8.62 ± 2.00	0.472	8.15 ± 1.74	8.88 ± 2.23	0.386	8.09 ± 1.76	9.00 ± 2.06	0.235
BMI, *n* (%)				0.356			1			0.466			0.924			0.322
Underweight	2 (4.8)	2 (10)	0 (0)		1 (4.3)	1 (5.3)		2 (5.9)	0 (0)		2 (5.9)	0 (0)		2 (6.1)	0 (0)	
Normal weight	18 (42.9)	10 (50)	8 (36.4)		10 (43.5)	8 (42.1)		16 (47.1)	2 (25.0)		15 (44.1)	3 (37.5)		12 (36.4)	6 (66.7)	
Overweight	8 (19)	3 (15)	5 (22.7)		4 (17.4)	4 (21.1)		5 (14.7)	3 (37.5)		6 (17.6)	2 (25.)		6 (18.2)	2 (22.2)	
Obese	14 (33.3)	5 (25)	9 (40.9)		8 (34.8)	6 (31.6)		11 (32.4)	3 (37.5)		11 (32.4)	3 (37.5)		13 (39.4)	1 (11.1)	

CPRS, Conners Parent Rating Scale; T-DSM-IV-S, Turgay DSM-based Disruptive Behavior Disorders Rating Scale; SD, standard deviation. BMI, body mass index. Gender and BMI were assessed using Fisher’s exact test. Age was assessed using the Mann–Whitney U test.

**Table 2:** Rare and common *SNAP-25* gene variants detected in children with ADHD.

ADHD Children							
**No**	Variant (HGVS cDNA)	dbSNP ID	Genomic Position (GRCh38)	Variant Type	ACMG Classification	Amino Acid Change	Highest Population MAF
1	SNAP25:c.-34C > A	rs11547872	chr20:10275458 C > A	5′UTR variant	Benign	–	0.03
2	SNAP25:c.72 + 41G > T	rs362585	chr20:10275604 G > T	intron variant	Benign	–	0.23
3	SNAP25:c.330C > T	rs362998	chr20:10296973 C > T	synonymous variant	Benign	p.Asp110=	0.25
4	SNAP25:c.552 + 23G > A	rs363006	chr20:10299435 G > A	intron variant	Benign	–	0.34
5	SNAP25:c.*54 T > C	novel	chr20:10306251 T > C	3′UTR variant	Not classified	–	NA

ADHD, attention-deficit/hyperactivity disorder; HGVS, Human Genome Variation Society; cDNA, complementary DNA; dbSNP, Single Nucleotide Polymorphism Database; GRCh38, Genome Reference Consortium Human Build 38; UTR, untranslated region; ACMG, American College of Medical Genetics and Genomics; AMP, Association for Molecular Pathology; MAF, minor allele frequency; ESP, Exome Sequencing Project; gnomAD, Genome Aggregation Database; chr, chromosome; NA, data not available. ACMG classification was performed according to the 2015 ACMG/AMP guidelines. The highest minor allele frequency observed in any population, including 1000 Genomes Phase 3, ESP, and gnomAD, was used. Gene variant, position, ACMG classification, amino acid change, and pathogenic criteria were taken from Franklin Bioinformatics. Variation type and the highest population MAF were taken from Ensembl. A dash (–) in the amino acid change column indicates “not applicable”. The notation p.Asp110= indicates that the aspartic acid residue at position 110 remains unchanged.

**Table 3:** *SNAP-25* gene variations in haplotype analysis.

Marker	Polymorphism	Allele Change	Contig Position	MAF
Marker 1	rs362585	G > T	10275604	0.095
Marker 2	rs362998	C > T	10296973	0.095
Marker 3	rs363006	G > A	10299435	0.107

MAF, minor allele frequency.

**Table 4:** Single and block haplotype analysis of variations in the *SNAP-25* gene.

Frequencies						
Marker	Haplotype	Overall	Non-Responders	Responders	Chi Square	*p* Value
M1	G		0.974	0.848	3.825	0.050
M2	C		0.974	0.848	3.825	0.050
M3	G		0.895	0.891	0.003	0.959
M1 + M2	GC	0.880	0.973	0.803	5.714	**0.016**
M1 + M2	TT	0.071	0.026	0.108	2.107	0.146
M1 + M2	TC	0.024	0.000	0.045	1.707	0.191
M1 + M2	GT	0.024	0.000	0.045	1.707	0.191
M1 + M3	GG	0.798	0.868	0.739	2.155	0.142
M1 + M3	GA	0.107	0.105	0.109	0.003	0.959
M1 + M3	TG	0.095	0.026	0.152	3.825	0.050
M2 + M3	CG	0.798	0.868	0.739	2.155	0.142
M2 + M3	CA	0.107	0.105	0.109	0.003	0.959
M2 + M3	TG	0.095	0.026	0.152	3.825	0.050
M1 + M2 + M3	GCG	0.773	0.868	0.694	3.577	0.058
M1 + M2 + M3	GCA	0.107	0.105	0.109	0.003	0.959
M1 + M2 + M3	TTG	0.071	0.026	0.108	2.104	0.146
M1 + M2 + M3	TCG	0.025	0.000	0.045	1.709	0.191
M1 + M2 + M3	GTG	0.025	0.000	0.045	1.709	0.191

Note: Bold values indicate statistically significant results (*p* < 0.05).

## Data Availability

The data that support the findings of this study are available upon reasonable request from the corresponding author. The data are not publicly available due to privacy or ethical restrictions. Restrictions apply to the availability of these data, which were used under license for this study.
